# Self-injurious behavior in Greek adolescents: the role of mental health problems and COVID-19 trauma

**DOI:** 10.1186/s12888-025-07040-7

**Published:** 2025-06-05

**Authors:** Georgios Giannakopoulos, Foivos Zaravinos-Tsakos, Efrosyni Pilafa, Andre Sourander, Gerasimos Kolaitis

**Affiliations:** 1 Department of Child Psychiatry, School of Medicine, National and Kapodistrian University of Athens, “Aghia Sophia” Children’s Hospital, Thivon and Papadiamantopoulou, Athens, 115 27 Greece; 2 School Of Medicine, National and Kapodistrian University of Athens, Athens, Greece; 3 Department of Child Psychiatry, Clinical Medicine, University of Turku, Turku, Finland

**Keywords:** Self-injurious behavior, Adolescence, Mental health problems, Intrusive symptoms, COVID-19 pandemic

## Abstract

**Background:**

Self-injurious behavior (SIB) in adolescents is a pressing public health issue, compounded by emotional dysregulation, behavioral challenges, and increased suicide risk. While much research has focused on interpersonal trauma, the impact of non-interpersonal traumatic events—such as those stemming from the COVID-19 pandemic—remains less clear.

**Methods:**

We investigated the associations between SIB, mental health difficulties, and COVID-19–related traumatic stress in a non-clinical sample of 5,612 Greek adolescents (55.4% female; mean age = 13.42 ± 0.96 years) from the Global Child and Adolescent Mental Health Study. Participants completed the Strengths and Difficulties Questionnaire (SDQ) to assess emotional symptoms, conduct problems, hyperactivity, and peer difficulties, and the Children’s Revised Impact of Event Scale-8 (CRIES-8) to evaluate post-traumatic stress symptoms. SIB was assessed via self-report, and specific COVID-19–related experiences (e.g., quarantine, hospitalization) were recorded alongside measures of suicidal ideation and suicide attempts.

**Results:**

Overall, 25.5% (*n* = 1,424) of adolescents reported engaging in SIB, 28.1% (*n* = 1,566) endorsed suicidal ideation, and 8.4% (*n* = 471) had attempted suicide. Adolescents reporting SIB had significantly higher total SDQ scores (M = 17.47 [SD = 5.82]) than those without (M = 11.22 [SD = 5.40]; *p* < 0.001). In logistic regression analyses, each one-point increase in emotional symptoms (OR = 1.17, 95% CI [1.12, 1.21]), conduct problems (OR = 1.17, 95% CI [1.12, 1.23]), and peer problems (OR = 1.08, 95% CI [1.03, 1.15]), as well as each one-point decrease in prosocial behavior (OR = 0.92, 95% CI [0.88, 0.96]), significantly elevated the odds of SIB. Moreover, higher intrusive symptoms on the CRIES-8 were modestly associated with increased odds of SIB (OR = 1.03, 95% CI [1.01, 1.05]). Among COVID-19–related experiences, personal hospitalization due to COVID-19 predicted SIB (OR = 1.26, 95% CI [1.04, 1.53]). Additionally, female gender (OR = 2.33, 95% CI [2.04, 2.63]), suicidal ideation (OR = 4.82, 95% CI [4.09, 5.69]), and a history of suicide attempts (OR = 5.08, 95% CI [3.77, 6.83]) further compounded the risk.

**Conclusions:**

Our findings demonstrate a multifaceted interplay between mental health difficulties and trauma-related stress in the emergence of SIB among adolescents. These data highlight the importance of early detection and targeted interventions addressing both emotional dysregulation and trauma-specific symptoms, particularly for youth with direct, severe COVID-19 experiences, to mitigate SIB and its associated risks.

## Introduction

Adolescence represents a critical developmental period marked by rapid physiological, cognitive, and socio-emotional changes. During this time, youths experience significant challenges related to identity formation, autonomy, and shifting interpersonal relationships [1]. These dynamic changes, while integral to maturation, can also render adolescents particularly vulnerable to mental health disturbances. Among the various behavioral manifestations of psychological distress, self-injurious behavior (SIB) has emerged as a significant public health concern, not only because of its high prevalence but also due to its association with increased risk of suicidal behavior [2, 3].

SIB, often operationalized as non-suicidal self-injury, is defined as the deliberate, self-inflicted damage to one’s own body tissue without the conscious intent to die [4]. For clarity and consistency, we use the term “SIB” throughout this manuscript. Although SIB is conceptually distinct from suicidal behavior, the two often coexist and share common risk factors, making differentiation challenging in both research and clinical practice [5, 6]. A recent meta-analysis [7] indicates that the aggregate lifetime prevalence of SIB in non-clinical adolescent samples is estimated at 22%. SIB onset typically occurs during early adolescence and peaking in mid-adolescence [8]. Furthermore, gender differences are well-documented, with females generally exhibiting higher rates of SIB than males [7, 9, 10].

Multiple theoretical frameworks have been proposed to explain the emergence and persistence of SIB. One of the most prominent perspectives is that SIB functions as an emotion regulation strategy [11]. Adolescents who experience intense, overwhelming negative emotions may resort to SIB as a maladaptive coping mechanism to alleviate their distress, effectively using physical pain to counterbalance emotional pain [12]. Alongside emotional dysregulation, externalizing behaviors such as conduct problems and hyperactivity have been identified as significant correlates of SIB, suggesting that impulsivity and behavioral disinhibition also play important roles [13, 14]. Moreover, difficulties in peer relationships and interpersonal functioning are frequently reported among self-injuring adolescents, highlighting the role of social stressors and isolation in exacerbating the risk for such behaviors [15, 16].

In addition to emotion regulation models, other theoretical frameworks have been proposed. The interpersonal theory posits that SIB serves to influence or communicate with others, such as eliciting care or demonstrating distress [17]. The social learning model emphasizes that SIB can be learned through exposure to others’ SIB or media representations [18]. Biological models suggest that abnormalities in pain perception or neurochemical functioning, particularly in the serotonergic system, may predispose individuals to engage in SIB [19]. Finally, experiential avoidance theories argue that SIB is used to escape or avoid unwanted internal experiences, such as distressing thoughts or memories [20].

In recent years, the global outbreak of the COVID-19 pandemic has introduced a novel set of challenges, profoundly impacting the mental health of adolescents across the world. The pandemic disrupted educational routines, social interactions, and family dynamics while exposing youths to non-interpersonal traumatic events such as quarantine, hospitalization, and bereavement [21]. These experiences have not only elevated levels of stress and anxiety but have also led to an increase in post-traumatic stress symptoms among children and adolescents [22]. Specifically, intrusive symptoms—characterized by recurrent, distressing memories and thoughts related to the traumatic event—have been implicated in the exacerbation of maladaptive coping strategies, including SIB [23–26].

Despite growing recognition of the pandemic’s impact on mental health, research focusing on the interplay between COVID-19–related traumatic stress and SIB in adolescents remains limited. While existing literature has robustly linked interpersonal trauma to SIB [27], the specific contributions of non-interpersonal traumatic events—such as those experienced during the COVID-19 crisis—warrant further investigation. Understanding whether direct experiences (e.g., hospitalization due to COVID-19) have a differential impact compared to more indirect exposures (e.g., quarantine or loss of a loved one) is crucial for tailoring effective intervention strategies.

Building on developmental models emphasizing emotion regulation vulnerabilities and neurodevelopmental changes during adolescence [12, 19], the present study aimed to clarify the unique role of non-interpersonal trauma in SIB. Specifically, we sought to examine whether COVID-19–related stress explained variance in SIB above and beyond general emotional and behavioral difficulties. By distinguishing the contributions of non-interpersonal trauma from broader mental health problems, we aimed to better understand the complex interplay between pandemic-related stress, underlying psychological vulnerabilities, and adolescent SIB.

The present study seeks to address these gaps by examining the relationship between SIB, general mental health problems, and COVID-19–related traumatic stress in a large sample of Greek adolescents. Drawing on data from the Global Child and Adolescent Mental Health Study (GCAMHS), our investigation is guided by several specific objectives: (1) To assess the association between SIB and a range of mental health difficulties, including emotional symptoms, conduct problems, hyperactivity, and peer problems, as measured by the Strengths and Difficulties Questionnaire (SDQ), (2) To investigate the contribution of COVID-19–related post-traumatic stress symptoms—with a particular focus on distinguishing between intrusive and avoidance symptoms—to the risk of SIB, using the Children’s Revised Impact of Event Scale-8 (CRIES-8), (3) To explore the impact of specific COVID-19–related experiences (e.g., quarantine, personal hospitalization, hospitalization of relatives or friends, and bereavement) on both trauma symptoms and SIB, and (4) To evaluate the role of gender and suicidality in compounding the risk of SIB, thereby clarifying the complex relationship between non-suicidal self-injury and suicidal ideation or attempts.

By integrating these dimensions, our study aims to provide a comprehensive and nuanced understanding of the multifactorial influences underlying SIB during a period of global crisis. This expanded perspective is critical for developing targeted, trauma-informed interventions that address both the general mental health challenges and the specific traumatic experiences that contribute to SIB among adolescents. Ultimately, the insights derived from this research have the potential to inform clinical practice and public health policy, fostering early identification and more effective prevention strategies for a behavior that poses significant risks to adolescent well-being.

## Methods

### Participants and procedure

This study is a cross-sectional investigation conducted as part of the multinational “Global Child and Adolescent Mental Health Study” (GCAMHS). The GCAMHS was the first systematic assessment of adolescent psychological distress, encompassing both general psychosocial adjustment and the post-traumatic impact of the COVID-19 pandemic. Data were collected between January 2023 and May 2023.

Of the adolescents targeted for participation, 54% (*n* = 6,108) completed the questionnaire. After applying inclusion criteria and removing incomplete surveys, the final analytic sample consisted of 5,612 adolescents. Adolescents were recruited from junior high schools (grades 7–9) across six regions of Greece (Attica, Thessaloniki, Kefalonia, Ilia, Karditsa, Tinos). Participants were eligible if they were between 12 and 16 years of age, had sufficient knowledge of the Greek language, and provided parental consent. Fifty-one schools were selected through a convenience sampling strategy to ensure geographic diversity by including urban, semi-urban, and rural areas. Within each participating school, all eligible students were invited to complete the anonymous paper-based survey during regular class hours under the supervision of trained research assistants. No randomization was applied within schools. Non-participation was primarily due to lack of parental consent (89.3%) or absenteeism during questionnaire administration (10.6%). Although basic demographic information (e.g., gender, SES, place of birth) was not systematically collected from non-participants, the possibility of selection bias cannot be excluded. It is plausible that non-participating adolescents, particularly those whose parents did not provide consent, may have differed systematically from participants in characteristics such as socioeconomic background or migration status. The study received ethical approval from both the Ministry of Education and Religious Affairs and Sports (Ref. No.: 4769/Δ2/17-01-2023).

### Measures

#### Demographic characteristics

Demographic data were collected on participants’ age, gender, grade, area of residence, family composition, economic status, and place of birth. Gender was reported as boy, girl, or non-binary, though only adolescents identifying as boys or girls were included in the analysis due to the small proportion (1.7%) identifying as non-binary. Family composition was dichotomized as living with biological parents versus other arrangements. Economic status was categorized as low, medium, or high based on participants’ self-assessment.

### Strengths and difficulties questionnaire (SDQ)

Mental health problems were assessed using the Strengths and Difficulties Questionnaire (SDQ) [28]. The self-report version, validated for use in Greece [29], includes 25 items divided into five scales: emotional symptoms, conduct problems, hyperactivity-inattention, peer problems, and prosocial behavior. Responses are scored as 0 (“not true”), 1 (“somewhat true”), or 2 (“certainly true”), with higher scores indicating worse mental health for the first four subscales and better mental health for the prosocial tendency subscale. The total SDQ scores, can be classified as falling in the normal r (0–16), borderline (17–18), and abnormal range (19–40) [29]. The internal consistency of the SDQ total difficulties score was good. Cronbach’s alpha was 0.79 based on standard assumptions, and the ordinal alpha (which accounts for the ordinal nature of the items) was also 0.79, indicating satisfactory reliability for research purposes [30]. We confirm that our study employed the paper version of the Strengths and Difficulties Questionnaire (SDQ). According to the Copyright Notice from Youthinmind Ltd, paper versions may be downloaded and photocopied at no charge for individuals or non-profit organizations, provided that no fee is charged to families. Accordingly, no separate license was obtained as the use of the paper version falls under these terms.

### Children’s revised impact of events scale (CRIES-8)

The Children’s Revised Impact of Events Scale-8 [31] was used to measure post-traumatic stress symptoms related to COVID-19. This cross-culturally validated self-report scale includes eight items rated on a four-point scale (0 = “not at all” to 5 = “often”) and has two subscales: intrusion and avoidance. A score of 17 has been identified as the clinical threshold for detecting PTSD [31]. The CRIES-8 demonstrated excellent internal consistency reliability in this sample (Cronbach’s α = 0.89).

### Experiences of COVID-19

Four closed-ended questions (Yes/No) assessed participants’ experiences related to COVID-19: (a) “Have you been quarantined by health authorities due to COVID-19?” (b) “Have you been hospitalized due to COVID-19?” (c) “Have any of your relatives or friends been hospitalized due to COVID-19?” (d) “Has any member of your family died due to COVID-19?”.

### SIB & suicidality

SIB was assessed through the self-report question: “Have you intentionally attempted to injure yourself, e.g., cut your skin?” Responses included “No, never”, “Yes, once”, “Yes, more than once”. For analysis, a dichotomous variable (Yes/No) was created to indicate any SIB experience. Previous studies have used similar single-item measures for SIB [32, 33]. Suicidality was assessed with two self-report questions: “Have you ever thought about ending your life?” (suicidal ideation) and “Have you tried to end your life?” (suicide attempt), with classification identical to that for SIB.

### Statistical analysis

Means *(M)* and standard deviations *(SD*) were reported for continuous data, while frequency distributions were used for categorical data. The relationship between SIB and demographic characteristics, suicidality, and COVID-19 pandemic-related stressful experiences was investigated using Chi-Square (χ²) tests for categorical variables and Independent Samples T-Tests for continuous variables. Independent samples t-tests were also conducted to explore differences in SDQ and CRIES-8 scores related to SIB. Alongside p-values, effect sizes were calculated to quantify the magnitude of group differences and associations. Cramer’s V was computed for Chi-Square tests, and Cohen’s d was calculated for independent samples t-tests. Further, hierarchical logistic regression analysis was conducted to identify factors significantly influencing SIB, with SIB as the dependent variable. Demographic factors (gender, age) were entered in Block 1 and psycho-social factors (i.e. SDQ subscales, CRIES-8 subscales, and stressful experiences during the COVID-19 pandemic) were entered in Block 2. Finally, suicidality related factors (suicidal ideation and suicidal attempts) were entered in Block 3. The enter method was used to include variables in the logistic regression model. Odds ratios (ORs) and 95% confidence intervals were presented. Variance Inflation Factors (VIFs) were calculated for the SDQ subscales to examine potential multicollinearity. All VIFs were below 2, indicating no significant multicollinearity. To address the potential for clustering effects due to the school-based sampling, we conducted a logistic mixed-effects model with a random intercept for schools and calculated the intraclass correlation coefficient (ICC). The ICC was 0.0006, indicating negligible between-school variability; therefore, single-level logistic regression was deemed appropriate. Additionally, model fit was assessed using the Hosmer-Lemeshow goodness-of-fit test, as well as the Akaike Information Criterion (AIC) and the Bayesian Information Criterion (BIC) indices. Exploratory interaction analyses were also conducted to examine whether gender moderated the associations between key psychosocial predictors (emotional symptoms, conduct problems, hyperactivity, peer problems, and intrusive symptoms) and SIB. Statistical tests were two-tailed and a p value of 0.05 or lower was considered statistically significant. Missing data were handled using listwise deletion, as the proportion of missing values was minimal (< 5%) across variables. All analyses of data were performed using SPSS (Version 29).

## Results

### Demographic characteristics

The final analytic sample included 5,612 adolescents (55.4% female) with a mean age of 13.42 years (SD = 0.96). Most participants (73.2%) resided in urban areas, and the vast majority (98.1%) were born in Greece. A large proportion of participants (86.5%) lived with both biological parents, and 68.1% reported a medium economic level, while 22.1% and 9.8% reported low and high economic levels, respectively (Table 1).

**Table 1:** Demographic characteristics and COVID-19 related experiences of adolescents by SIB status

Variables	Total (*N* = 5612)		SIB (*N* = 1424)		Non-SIB (*N* = 4164)					
	*N*	%	*N*	%	*N*	%	M	SD	χ² /t	Cramer’s V
**Gender**									168.71***	0.219
Female	3110	55.4	1001	17.9	2102	37.6				
Male	2502	44.6	423	7.6	2062	36.9				
**Age**							13.42	0.96	-2.50*	—
**Grade**									6.77*	0.028
7	1867	33.4	435	7.8	1419	25.5				
8	1847	33	499	9	1340	24.1				
9	1878	33.6	485	8.7	1390	25				
**Area of residence**									7.30**	0.038
Urban	4106	73.2	1081	19.3	3008	53.8				
Rural	1506	26.8	343	6.1	1156	20.7				
**Born in Greece**									9.68**	0.041
Yes	5491	98.1	1384	24.8	4084	73.3				
No	105	1.9	34	0.6	71	1.3				
**Live with two biological parents**									73.89***	0.119
Yes	4851	86.5	1137	20.4	3697	66.2				
No	756	13.5	287	5.1	464	8.3				
**Financial Status**									1.76	0.000
Low	1222	22.1	325	5.9	894	16.2				
Medium	3771	68.1	954	17.3	2803	50.8				
High	545	9.8	128	2.3	411	7.5				
**Quarantine**									4.24*	0.028
Yes	4,454	80	1,163	21.0	3282	59.2				
No	1,113	20	255	4.6	847	15.3				
**Hospitalization**									28.01***	0.072
Yes	850	15.3	278	5.0	569	10.2				
No	4722	84.7	1,139	20.5	3566	64.2				
**Hospitalization of relatives/friends**									34.33***	0.078
Yes	2782	50	803	14.5	1968	35.5				
No	2781	50	613	11.1	2159	39.0				
**Death of family member**									33.90***	0.081
Yes	664	11.9	230	4.1	431	7.8				
No	4903	88.1	1186	21.4	3700	66.7				

Cramer’s V values are reported as effect size estimates for Chi-square tests

*p < 0.05, **p < 0.01, ***p < 0.001

### SIB and suicidality

Of the participants, 25.5% (*n* = 1,424) reported having intentionally injured themselves at least once. Additionally, 28.1% (*n* = 1,566) reported experiencing suicidal ideation, and 8.4% (*n* = 471) had attempted suicide at least once.

### Mental health problems

The mean scores on the SDQ subscales for the total sample were as follows: Emotional Symptoms (M = 3.29, SD = 2.63), Conduct Problems (M = 2.76, SD = 1.82), Hyperactivity (M = 4.84, SD = 1.63), Peer Problems (M = 4.34, SD = 1.70), and Prosocial Behavior (M = 7.70, SD = 1.95). The overall SDQ score for the sample averaged 12.81 (SD = 6.15). Adolescents who engaged in SIB had significantly higher total SDQ scores (M = 17.47, SD = 5.82) compared to those who did not engage in SIB (M = 11.22, SD = 5.40), t(5586) = 35.59, *p* < 0.001, d = 1.11 (Table 2). Large effect sizes were also observed for emotional symptoms (d = 0.98) and conduct problems (d = 0.75). When categorizing the total SDQ scores, 59.3% of the adolescents fell within the normal range (0–16), 10.2% were in the borderline range (17–18), and 30.5% were classified as abnormal (19–40). These categorizations further underscore the association between higher SDQ scores and SIB, with a significant proportion of adolescents with SIB falling into the abnormal range.

**Table 2:** Comparison of mental health and post-traumatic stress scores between adolescents with and without SIB

	Total (*N* = 5612)		SIB (*N* = 1424)		Non-SIB (*N* = 4164)			
	M	SD	M	SD	M	SD	t	Cohen’s d
**SDQ**								
Emotional symptoms	3.29	2.63	5.06	2.68	2.69	2.33	-31.80***	0.98
Conduct problems	2.76	1.82	4.34	1.95	2.43	1.68	-23.91***	0.75
Hyperactivity	4.84	1.63	5.38	1.72	4.65	1.57	-14.05***	0.45
Peer problems	4.34	1.70	4.70	1.51	4.21	1.22	-10.98***	0.37
Prosocial behavior	7.70	1.95	7.55	2.06	7.76	1.91	3.48***	-0.13
Total score	12.81	6.15	17.47	5.82	11.22	5.40	-35.59***	1.11
**CRIES-8**								
Intrusion	3.67	4.67	5.43	5.57	3.08	4.16	-16.79***	0.52
Avoidance	4.98	5.96	6.54	6.39	4.46	5.71	-11.48***	0.35
Total score	8.66	9.67	11.97	10.92	7.54	8.93	-15.21***	0.47

Cohen’s *d* values are reported as effect size estimates for t-tests

*p < 0.05, **p < 0.01, ***p < 0.001

### COVID-19 related experiences and post-traumatic stress symptoms

The prevalence of adolescents with a high probability of being diagnosed with PTSD, as indicated by a CRIES-8 score above the clinical threshold of 17, was 21.4%. Specifically, 30.5% of adolescents who were quarantined, 35.8% of those hospitalized, 37.2% of those with a hospitalized relative or friend, and 43.6% of those who experienced the death of a family member due to COVID-19 met the criteria for probable PTSD. Adolescents who were quarantined due to COVID-19 had significantly higher CRIES-8 scores (M = 9.3, SD = 9.78) compared to those who were not quarantined (M = 7.33, SD = 9.20), t(5565) = -5.24, *p* < 0.001, d = 0.21. Similarly, those hospitalized due to COVID-19 had higher CRIES-8 scores (M = 10.56, SD = 10.37) compared to those not hospitalized (M = 8.34, SD = 9.51), t(5570) = -6.15, *p* < 0.001, d = 0.22. Additionally, adolescents with relatives or friends hospitalized due to COVID-19 reported higher CRIES-8 scores (M = 10.37, SD = 10.10) compared to those without such experiences (M = 7.03, SD = 8.95), t(5561) = -13.01, *p* < 0.001, d = 0.36. Finally, those who lost a family member due to COVID-19 exhibited significantly higher CRIES-8 scores (M = 13.58, SD = 10.96) than those who did not experience such a loss (M = 8.02, SD = 9.30), t(5565) = -14.14, *p* < 0.001, d = 0.48 (Table 3).

**Table 3:** Impact of COVID-19 related experiences on post-traumatic stress scores in adolescents

CRIES-8	M	SD	t	Cohen’s d
**Quarantine due to COVID-19**			5.24***	0.21
Yes	9.3	9.78		
No	7.33	9.20		
**Hospitalization due to COVID-19**			6.15***	0.22
Yes	10.56	10.37		
No	8.34	9.51		
**Hospitalization of relatives/friends due to COVID-19**			13.01***	0.36
Yes	10.37	10.10		
No	7.03	8.95		
**Death of family member due to COVID-19**			14.14***	0.48
Yes	13.58	10.96		
No	8.02	9.30		

Cohen’s *d* values are reported as effect size estimates for t-tests. Effect sizes are based on available data. *p < 0.05, **p < 0.01, ***p < 0.001

### Role of mental health problems, COVID-19 related experiences, post-traumatic stress symptoms, gender and suicidality in SIB

The hierarchical logistic regression analysis (Table 4) assessed the contribution of demographic, psycho-social and suicide related factors to SIB. Model diagnostics indicated a significant Hosmer-Lemeshow goodness-of-fit test in the final model (*p* = 0.002), suggesting some deviation from perfect model fit. However, model evaluation based on additional fit indices, including acceptable AIC (4703.23) and BIC (4840.45) values, supported the adequacy of the model. Among the demographic factors (Block 1), gender indicated a positive association with SIB. Specifically, males were 47% less likely than females to engage in SIB (OR = 0.43, 95% CI [0.38, 0.49]). The psycho-social factors (Block 2) resulted in a significant improvement in the prediction of SIB. Higher scores on the emotional symptom subscale were associated with a 28% increase in the likelihood of SIB for each one-point increase (OR = 1.28, 95% CI [1.24, 1.32]). Similarly, each one-point increase in the conduct problems subscale was linked to a 23% increase in the likelihood of SIB (OR = 1.23, 95% CI [1.22, 1.32]), and each one-point increase in peer problems was associated with a 14% increase in the odds of SIB (OR = 1.14, 95% CI [1.08, 1.20]). Additionally, each one-point decrease in prosocial behavior was associated with a 10% increase in the odds of SIB (OR = 0.90, 95% CI [0.87, 0.93]). Intrusive symptoms also showed a significant association, with a 4% increase in SIB odds per point increase (OR = 1.04, 95% CI [1.02, 1.06]). After adjusting for demographic and psycho-social factors, suicide related factors, the presence of suicide related factors (Block 3) significantly improved the prediction of SIB. Specifically, adolescents who reported suicidal ideation were 4.82 times more likely to engage in SIB compared to those without such thoughts (OR = 4.82, 95% CI [4.09, 5.69]), and those with a history of suicide attempts were 5.08 times more likely to engage in SIB (OR = 5.08, 95% CI [3.77, 6.83]).

**Table 4:** Hierarchical regression analysis predicting SIB in adolescents

Variables	Block 1		Block 2		Block 3	
	OR	95% CI	OR	95% CI	OR	95% CI
Demographic factors	0.84*	0.70–1.00	0.84*	0.70–1.00	0.84*	0.70–1.00
Gender	0.43***	0.38–0.49	0.73***	0.62–0.86	0.80*	0.67–0.96
Age	1.08*	1.01–1.15	1.03	0.96–1.11	0.98	0.90–1.06
Psycho-social factors						
Quarantine due to COVID-19			0.99	0.83–1.18	1.09	0.89–1.32
Hospitalisation due to COVID-19			1.26*	1.04–1.53	1.11	0.89–1.37
Hospitalisation of relatives/friends due to COVID-19			0.96	0.83–1.12	0.93	0.78–1.09
Death of family member due to COVID-19			1.16	0.95–1.44	1.08	0.86–1.37
SDQ Emotional symptoms			1.28***	1.24–1.32	1.17***	1.12–1.21
SDQ Conduct problems			1.23***	1.22–1.32	1.17***	1.12–1.23
SDQ Hyperactivity			1.00	0.96–1.05	1.03	0.98–1.09
SDQ Peer problems			1.14***	1.08–1.20	1.08**	1.03–1.15
SDQ Prosocial Behavior			0.90***	0.87–0.93	0.92***	0.88–0.96
CRIES– 8 Intrusion			1.04***	1.02–1.06	1.03**	1.01–1.05
CRIES– 8 Avoidance			1.00	0.99–1.01	0.99	0.98–1.01
Suicidality related factors						
Suicidal ideation					4.82***	4.09–5.69
Suicide attempt					5.08***	3.77–6.83
-2 log likelihood ratio	6061.26		5109.85		4370.76	
χ^2^ (df)	177.53 (2)***		1128.93 (13)***		1868.01 (15)***	
R_N_^2^	0.05		0.27		0.42	
HL	0.10		0.31		0.002	
AIC	–		–		4703.23	
BIC	–		–		4840.45	

OR = Odds Ratio; CI = Confidence Interval; df = Degrees of Freedom; SDQ = Strengths and Difficulties Questionnaire; CRIES = Children’s Revised Impact of Events Scale; R_N_^2^ = Nagelkerke’s pseudo R-squared; HL = Hosmer-Lemeshow goodness-of-fit p-value; AIC = Akaike Information Criterion; BIC = Bayesian Information Criterion

*p < 0.05, **p < 0.01, ***p < 0.001

While the descriptive analysis showed positive associations between stressful pandemic experiences (e.g., quarantine, hospitalization, loss of a loved one) and SIB (Table 1), only personal hospitalization due to COVID-19 significantly predicted SIB after controlling for other variables in the logistic regression model (OR = 1.26, 95% CI [1.04, 1.53]; Table 4).

Additional exploratory analyses examined whether gender moderated the associations between key psychosocial predictors (emotional symptoms, conduct problems, hyperactivity, peer problems, prosocial behavior, and intrusive symptoms) and SIB. None of the interaction terms reached statistical significance (all p-values > 0.05), although the interaction between gender and conduct problems approached significance (*p* = 0.075).

## Discussion

This study aimed to elucidate the intricate relationships among SIB, broader mental health problems, and the traumatic impact of the COVID-19 pandemic in a large sample of Greek adolescents. Our findings confirm that SIB is a prevalent concern, with over 25% of participants reporting having engaged in such behavior. Importantly, the results demonstrate that elevated emotional symptoms, conduct problems, and peer difficulties, as well as lower prosocial behavior, are all significantly associated with SIB. Moreover, intrusive post-traumatic symptoms related to COVID-19 emerged as a notable risk factor—even after accounting for multiple demographic and psychosocial variables—underscoring the potent role of trauma in the manifestation of SIB.

Adolescence is characterized by heightened emotional reactivity and challenges in emotion regulation [1]. Consistent with previous research [11, 12], our findings support the notion that SIB often functions as a maladaptive strategy for managing intense negative emotions. The strong association between higher scores on the emotional symptom subscale of the SDQ and SIB suggests that adolescents who struggle with emotional regulation are particularly vulnerable. This aligns with the conceptualization of SIB as a means of alleviating overwhelming affective states [4], and further underscores the need for interventions that enhance adaptive emotional coping skills.

Beyond emotional distress, behavioral issues, such as conduct problems and lower prosocial behavior, were robustly associated with SIB. These findings are consistent with prior studies that highlight the role of impulsivity and behavioral dysregulation in SIB [13, 14]. Moreover, the significant link between peer difficulties and SIB supports existing literature suggesting that interpersonal stressors, including problems in peer relationships, may exacerbate the risk of SIB [27]. Such challenges in social functioning may not only increase feelings of isolation but also diminish the availability of positive support, thus reinforcing maladaptive behaviors.

The COVID-19 pandemic introduced a unique set of stressors that appear to have had a lasting impact on adolescent mental health. While various pandemic-related experiences (e.g., quarantine, loss of loved ones) were associated with elevated post-traumatic stress scores, only personal hospitalization due to COVID-19 remained a significant predictor of SIB in the multivariate model. This finding suggests that direct exposure to severe illness may be associated with intensified trauma-related symptoms and increased rates of SIB. The significant association between intrusive symptoms and SIB echoes earlier research indicating that persistent, distressing memories and intrusive thoughts can undermine effective coping mechanisms [34]. In this context, the COVID-19 pandemic may have been associated with compounded vulnerabilities in adolescents already at risk due to emotional and behavioral dysregulation.

Intrusive symptoms may contribute to SIB through multiple, potentially overlapping mechanisms. One possibility is that the repeated re-experiencing of distressing events overwhelms adolescents’ emotional regulation capacities, thereby indirectly increasing the risk of SIB as a maladaptive coping strategy [35, 36]. In this framework, emotional dysregulation would mediate the relationship between intrusive symptoms and SIB. Alternatively, intrusive memories may trigger acute emotional distress and dissociative states, prompting more immediate and direct engagement in SIB to manage overwhelming internal experiences [37, 38]. Future research using longitudinal or experimental designs is needed to disentangle these potential pathways and clarify whether emotional dysregulation serves as a mediator or whether intrusive symptoms exert more immediate effects on behavior.

Our study further highlights the complex interplay between SIB and suicidality. Adolescents reporting suicidal ideation and attempts were significantly more likely to engage in SIB, reinforcing previous findings that indicate a close relationship between SIB and suicidal behaviors [5, 6]. Furthermore, exploratory analyses indicated that the relationships between psychosocial predictors and SIB were generally consistent across genders. Given that SIB is both a potential precursor to and co-occurring phenomenon with suicidal behavior, these results underscore the critical importance of comprehensive risk assessments in both clinical and educational settings. Early identification of SIB, particularly in the context of emerging suicidal ideation, may be key to preventing more severe outcomes.

The high prevalence of SIB, coupled with its robust associations with emotional dysregulation, behavioral problems, and trauma-related symptoms, has important implications for both clinical practice and public health policy. Routine screening for emotional and behavioral difficulties in schools and pediatric settings could facilitate early detection of at-risk adolescents. Importantly, screening efforts should extend beyond general emotional distress to include the identification of trauma-related symptoms, such as intrusive memories. Brief validated tools could be feasibly incorporated into mental health screenings conducted in schools and pediatric primary care settings. Teachers, school psychologists, and general practitioners could be trained to administer short trauma screening questionnaires or to recognize behavioral indicators of post-traumatic stress, including dissociation, hypervigilance, and emotional numbing. Early identification of adolescents experiencing high levels of intrusive symptoms may facilitate targeted referrals to trauma-focused interventions before maladaptive coping strategies, such as SIB, become entrenched.

Beyond screening, educational policy initiatives should prioritize mental health training for school personnel to increase awareness of trauma-related symptoms and to strengthen early intervention efforts. Integrating basic trauma-informed care principles into teacher training curricula and school mental health protocols could enhance the capacity of schools to support adolescents at risk for SIB and other trauma-related difficulties. Interventions aimed at improving emotion regulation, reducing impulsivity, and enhancing interpersonal skills may be particularly effective in mitigating the risk of SIB [39]. Furthermore, trauma-informed care approaches that specifically address intrusive post-traumatic symptoms could provide additional benefits, especially in the aftermath of large-scale crises such as the COVID-19 pandemic [40].

Several limitations of the current study warrant discussion. First, the cross-sectional design precludes the determination of causality among mental health problems, trauma-related symptoms, and SIB. Longitudinal research is needed to clarify the temporal sequencing and potential causal mechanisms underlying these associations. Second, the reliance on self-report measures, including the use of single-item questions to assess SIB, suicidal ideation, and suicide attempts, poses certain limitations. Single-item measures may lack the full construct validity of multi-item scales and do not capture important behavioral context such as the frequency, methods, motivations, and intent behind the behaviors. Although single-item measures are often necessary in large epidemiological surveys for feasibility reasons, future research would benefit from more comprehensive assessments that provide a richer understanding of the complexity of these behaviors. Third, the potential for selection bias must be considered, as we were unable to compare participants and non-participants on key demographic characteristics. Non-participants may have differed systematically on variables such as socioeconomic status or migration background, which could limit the generalizability of the findings. Finally, although our sample was large and geographically diverse within Greece, caution is advised when generalizing these findings to adolescents in different cultural or clinical contexts.

In conclusion, our study demonstrates that SIB among adolescents is multifactorially determined by emotional dysregulation, behavioral and interpersonal problems, and trauma-related distress stemming from the COVID-19 pandemic. The differential impact of direct versus indirect pandemic experiences suggests that the severity and personal relevance of traumatic events are critical factors in the emergence of SIB. These findings highlight the urgent need for early detection and comprehensive, multifaceted intervention strategies that address both underlying mental health challenges and trauma-specific symptoms. Future research could test these pathways using longitudinal mediation models to clarify whether emotional dysregulation mediates the relationship between intrusive trauma symptoms and the development of SIB over time. Such integrated approaches are essential not only for reducing SIB but also for mitigating the broader risk of suicidality among vulnerable adolescents.

## Data Availability

The datasets used and/or analysed during the current study are available from the corresponding author on reasonable request.
